# Managing eye injuries

**Published:** 2015

**Authors:** Dorothy Mutie, Nyawira Mwangi

**Affiliations:** Consultant Ophthalmologist: Jaramogi Oginga Odinga Teaching and Referral Hospital, Kisumu, Kenya. Email: **d_mutie@yahoo.co.uk**; Principal Lecturer: Ophthalmology Programmes, Kenya Medical Training College, Nairobi, Kenya. Email: **nyawiramwangi@yahoo.com**

**Figure:**
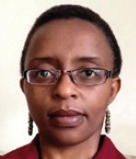
Dorothy Mutie

**Figure:**
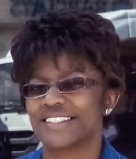
Nyawira Mwangi

Based on what you found during the eye examination (see page 46), classify the injury as a non-mechanical injury (chemical or thermal injury), a non-globe injury (orbital or adnexal injury) or as a mechanical globe injury.

In the case of mechanical globe injuries, it is important to classify the injury according to the Birmingham Eye Trauma Terminology System (BETTS) (page 43) and write it down in the patient's notes; this will help to ensure that everyone involved in caring for the patient will have a consistent understanding of the type of injury. The resulting uniformity of terminology also helps with research, making it possible to compare data and do audits of injuries – which is essential for prevention.

## Mechanical globe injury: open globe

### Ruptured globe

This presents with lowered intra-ocular pressure or flat anterior chamber, bloody chemosis and irregular pupil. Intra-ocular contents may be visible outside the globe.

**Treatment:** Immediately protect the eye with a plastic/metal shield and give tetanus toxoid. **Refer very urgently.**

### Penetrating injury

In this injury an entrance wound can be identified on the globe.

**Treatment:** Immediately protect the eye with a plastic/metal shield,

**Refer urgently.** Do not instil medication into the eye.

**Figure 1.**
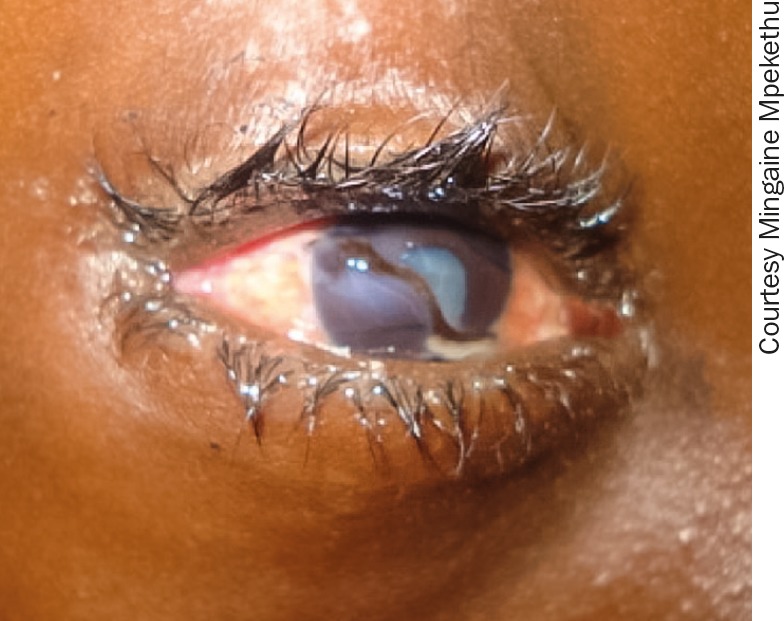
Penetrating corneal laceration, uvea in wound, traumatic cataract.

### Perforating injury

Presents with haemorrhagic chemosis, shallow anterior chamber, hyphaema, irregular pupil, poor view of the fundus and a positive Siedel's test.

**Treatment:** Immediately protect the eye with a plastic/metal shield. **Refer very urgently.**

### Intraocular foreign body (IOFB)

Should be suspected where there is history of trauma from flying objects (typically hitting metal with a hammer) and no external FB is found and/or where an entrance wound is noted.

**Treatment**

- Protect the eye with a hard eye shield (e.g. a metal eye shield) to protect the globe from external pressure.
- Give systemic analgesics.
- Give tetanus toxoid.
- An orbital X-ray, ocular ultrasound or CT scan is necessary.

**Refer very urgently.**

## Mechanical globe injury: closed globe

These are all partial-thickness wounds of the eyewall, i.e. the sclera and cornea.

### Contusion

Patients can present with chemosis, hyphaema and irregular pupil following blunt injury.

**Treatment:** Immediately protect the eye with a plastic/metal shield. Patients with hyphaema should be positioned with the head raised 30–45 degrees.

**Refer urgently.**

### Lamellar laceration

A partial thickness corneal or scleral laceration requires repair, unless it is already self-sealing.

**Treatment:** Primary closure (repair of laceration) using 10/0 nylon or silk. Pad the eye for 24 hours and give topical and systemic antibiotics.

If repair is not possible, **refer very urgently.**

### Foreign body on the surface of the eye

Wash any loose foreign bodies away by irrigating the eye.

For a corneal foreign body (FB):

- Anaesthetise the eye
- Remove the FB using the corner of a clean piece of paper, a Kimura spatula or the tip of a sterile 25G needle. Be **very** careful not to worsen the abrasion.
- Remove the rust left by a metallic FB.
- Evert the upper eyelid to look for additional FBs.
- Instil antibiotic ointment (erythromycin or tetracycline).
- Pad the eye.

**Figure 2.**
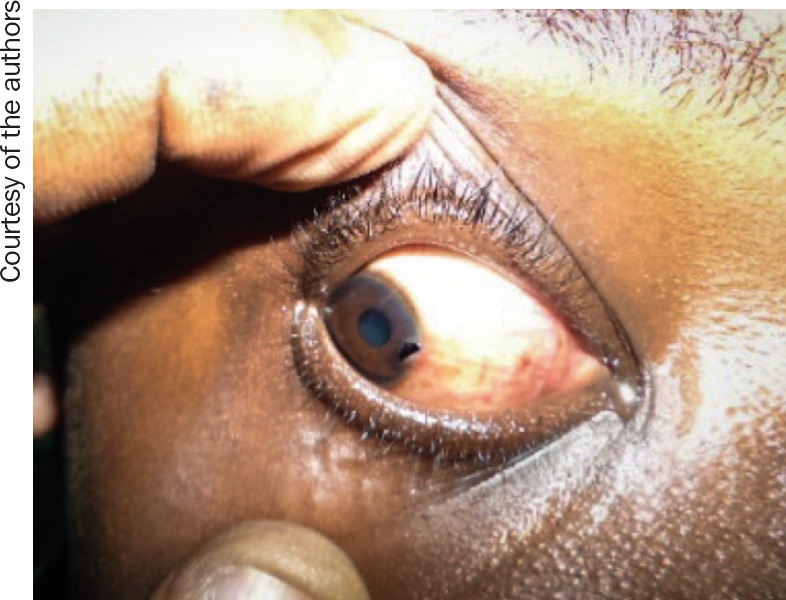
Corneal foreign body

**Review in 24 hours.**

## Chemical injuries

Alkali injuries may be caused by household bleach or ammonia-containing products, fertiliser, cement (lime) and fireworks (magnesium hydroxide). Acid injuries may be sustained from battery acid, nail polish remover (acetic acid) or vinegar.

**Figure 3.**
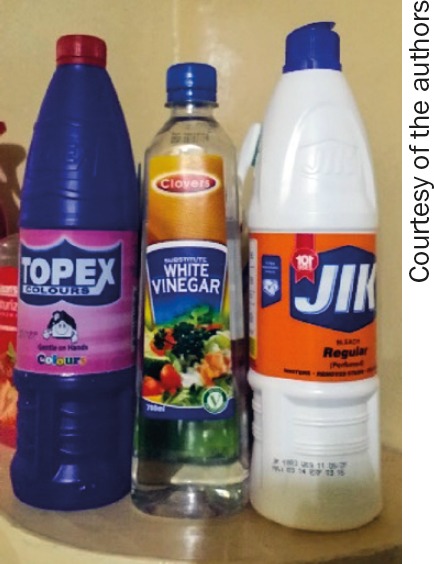
Household chemicals that cause ocular injuries

**Immediately, perform high volume-irrigation of the eye affected using ringer's lactate or normal saline (or clean tap water if these fluids are not available).**

**Figure 4.**
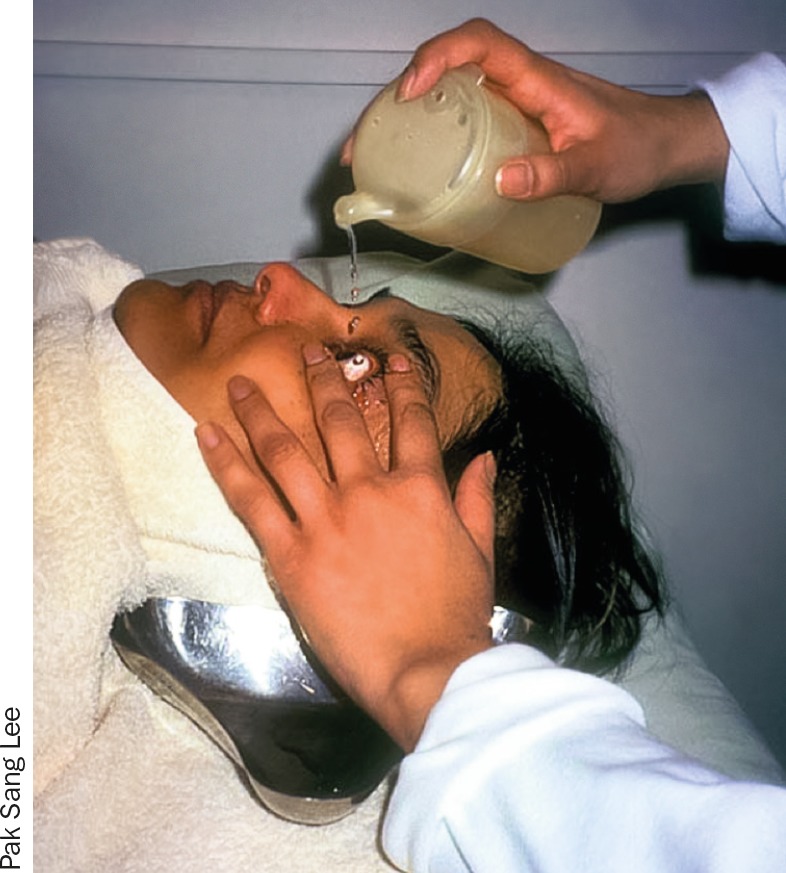
Irrigating the eye

- Instil topical anaesthetic.
- Insert lid speculum or use your fingers to gently hold the eyelids open (Figure 2).
- Irrigate one eye at a time.
- Position the patient's head sideways. Place a kidney dish ready to receive the fluid and towels to enhance patient comfort. Ideally, use an IV giving set at full speed. However, in the absence of this, a clean container may be used as shown in Figure 4.
- Irrigate with normal saline or Ringer's lactate for at least 30 minutes, taking care not to contaminate the unaffected eye.
- Evert the lid and irrigate the fornices.
- At intervals, check the pH. The target pH is 7.0–7.3.
- Instil cycloplegic drops and topical antibiotic eyedrops and pad the eye.
- Give systemic analgesics.

**Refer immediately.**

### Superglue in the eye

Remove clumps of glue from the eyelashes, apply a warm compress, instil antibiotic ointment and refer for further assessment. If the eyelids are firmly stuck together, do not try to prise them apart as they will eventually separate on their own.

**Figure 5.**
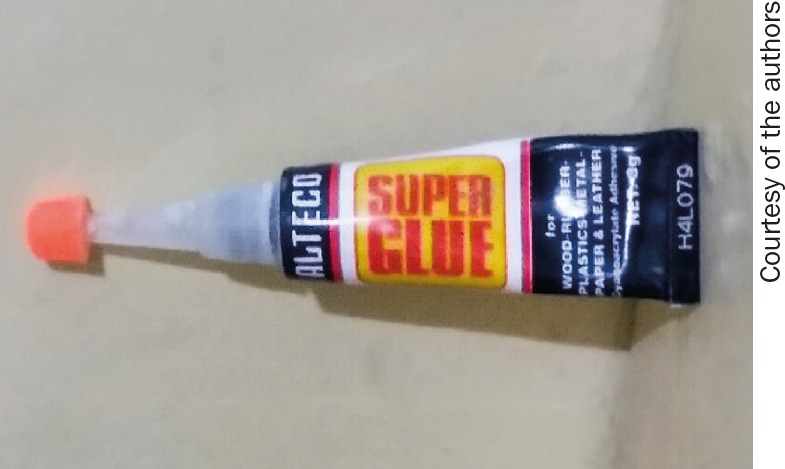
Tube of cyanoacrylate (superglue)

## Thermal ocular injuries

These are often sustained as a result of falling into fires or being splashed by hot fluids like boiling water or porridge. They can also be sustained from cigarettes and curling irons.

**Treatment:** Treat these injuries in the same way as chemical injuries, except that irrigation is not needed. Debridement of necrotic tissue may be required. Antibiotic ointment is instilled into the eye, and then the eyes are covered with a moist sterile dressing.

**Refer immediately.**

**Burns caused by light** can occur upon exposure to direct sunlight, observing an eclipse without protection, laser burns and infrared light exposure.

**Treatment:** Cover the eye with a sterile pad and eye shield

**Refer immediately.**

## Adnexal and orbital injuries

### Lid lacerations

Human or animal bites require especially rigorous cleaning, removal of devitalised tissue, and prophylactic systemic antibiotics. Anti-snake venom and rabies post-exposure prophylaxis should be administered, according to recommendations, for snake and dog bites respectively.

**Treatment:** Clean the wound thoroughly and remove foreign bodies while keeping debridement to a minimum. Handle tissues with care, and carefully align the anatomy before repairing using 6/0 sutures. Close the wound in layers.

**Refer very urgently.**

**Figure 6.**
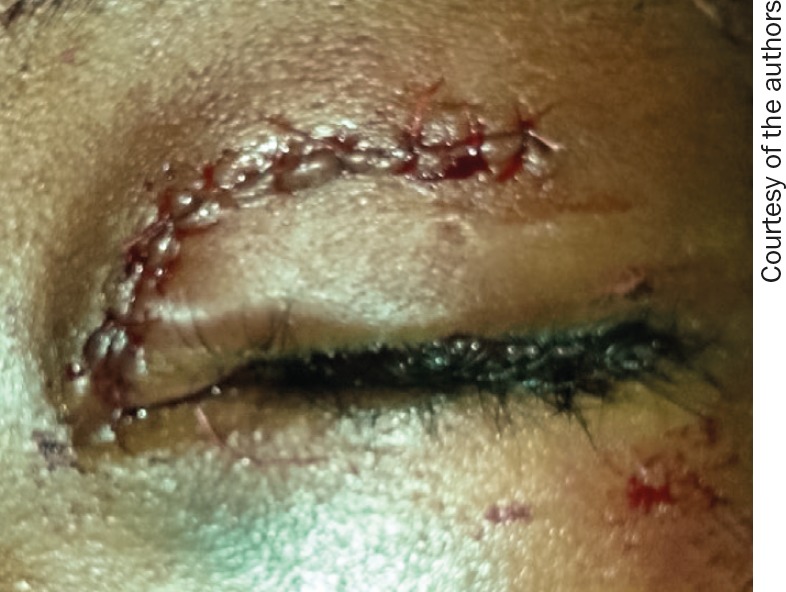
Careful alignment of anatomy

**Figure 7.**
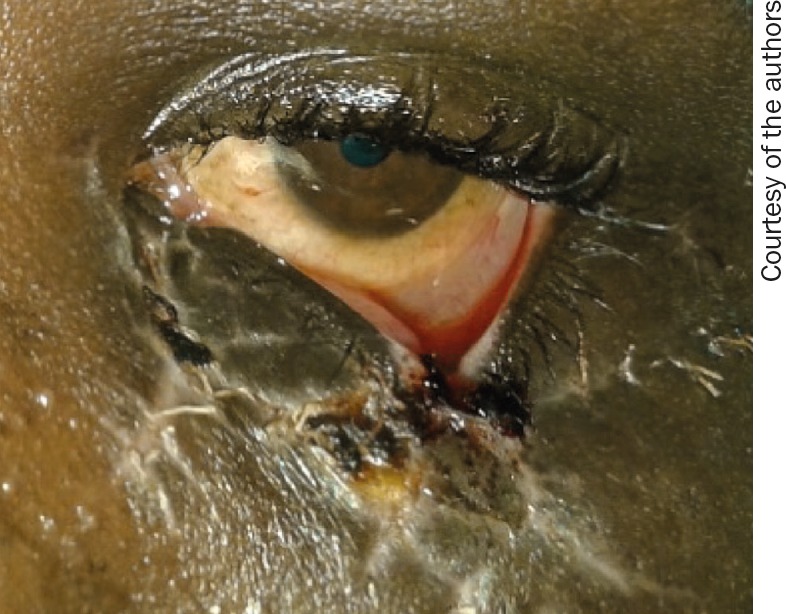
Poorly repaired lid lacerations lead to notches and inadequate closure of the lids

### Orbital fractures

These may be suspected when the presentation includes double vision, eyelid swelling after nose-blowing, peri-orbital subcutaneous emphysema, peri-ocular ecchymosis and oedema, ophthalmoplegia and enophthalmos. A plain X-ray of the orbit (AP and lateral views) and CT scan of the orbit would be useful.

**Refer urgently.**

### Retrobulbar haemorrhage

This presents with pain, proptosis, RAPD and increased IOP.

**Treatment:** Apply an eye shield.

**Refer very urgently.**

